# Non-invasively predicting euploidy in human blastocysts via quantitative 3D morphology measurement: a retrospective cohort study

**DOI:** 10.1186/s12958-024-01302-x

**Published:** 2024-10-28

**Authors:** Guanqiao Shan, Khaled Abdalla, Hang Liu, Changsheng Dai, Justin Tan, Junhui Law, Carolyn Steinberg, Ang Li, Iryna Kuznyetsova, Zhuoran Zhang, Clifford Librach, Yu Sun

**Affiliations:** 1 Department of Mechanical and Industrial Engineering, University of Toronto, Toronto, ON M5S 3G8 Canada; 2 CReATe Fertility Centre, Toronto, ON M5G 1N8 Canada; 3 School of Mechanical Engineering, Dalian University of Technology, Dalian, 116024 China; 4 Department of Computer Science, University of Toronto, Toronto, ON M5S 2E4 Canada; 5 School of Science and Engineering, The Chinese University of Hong Kong Shenzhen, Shenzhen, 518172 China; 6 Department of Obstetrics and Gynecology, University of Toronto, Toronto, ON M5G 1E2 Canada; 7 Sunnybrook Research Institute, Toronto, ON M4N 3M5 Canada

**Keywords:** Blastocyst, Euploidy prediction, 3D morphology measurement, Machine learning

## Abstract

**Background:**

Blastocyst morphology has been demonstrated to be associated with ploidy status. Existing artificial intelligence models use manual grading or 2D images as the input for euploidy prediction, which suffer from subjectivity from observers and information loss due to incomplete features from 2D images. Here we aim to predict euploidy in human blastocysts using quantitative morphological parameters obtained by 3D morphology measurement.

**Methods:**

Multi-view images of 226 blastocysts on Day 6 were captured by manually rotating blastocysts during the preparation stage of trophectoderm biopsy. Quantitative morphological parameters were obtained by 3D morphology measurement. Six machine learning models were trained using 3D morphological parameters as the input and PGT-A results as the ground truth outcome. Model performance, including sensitivity, specificity, precision, accuracy and AUC, was evaluated on an additional test dataset. Model interpretation was conducted on the best-performing model.

**Results:**

All the 3D morphological parameters were significantly different between euploid and non-euploid blastocysts. Multivariate analysis revealed that three of the five parameters including trophectoderm cell number, trophectoderm cell size variance and inner cell mass area maintained statistical significance (*P* < 0.001, aOR = 1.054, 95% CI 1.034–1.073; *P* = 0.003, aOR = 0.994, 95% CI 0.991–0.998; *P* = 0.010, aOR = 1.003, 95% CI 1.001–1.006). The accuracy of euploidy prediction by the six machine learning models ranged from 80 to 95.6%, and the AUCs ranged from 0.881 to 0.984. Particularly, the decision tree model achieved the highest accuracy of 95.6% (95% CI 84.9-99.5%) with the AUC of 0.978 (95% CI 0.882–0.999), and the extreme gradient boosting model achieved the highest AUC of 0.984 (95% CI 0.892-1.000) with the accuracy of 93.3% (95% CI 81.7-98.6%). No significant difference was found between different age groups using either decision tree or extreme gradient boosting to predict euploid blastocysts. The quantitative criteria extracted from the decision tree imply that euploid blastocysts have a higher number of trophectoderm cells, larger inner cell mass area, and smaller trophectoderm cell size variance compared to non-euploid blastocysts.

**Conclusions:**

Using quantitative morphological parameters obtained by 3D morphology measurement, the decision tree-based machine learning model achieved an accuracy of 95.6% and AUC of 0.978 for predicting euploidy in Day 6 human blastocysts.

**Trial registration:**

N/A.

**Supplementary Information:**

The online version contains supplementary material available at 10.1186/s12958-024-01302-x.

## Background

 During in vitro fertilization (IVF), embryo evaluation is a critical step to select embryos with high reproductive potential for transfer. Embryo aneuploidy is one of the leading causes of implantation failure [1, 2] and miscarriages [3, 4]. Preimplantation genetic testing for aneuploidy (PGT-A) is widely employed to determine embryo ploidy status. Due to the invasive nature of the biopsy procedure and the financial burden for genetic testing, whether PGT-A should be routinely used in IVF treatment is still under debate [5, 6]. Blastocoel fluid sampling (BFS) and spent blastocyst media (SBM) testing were developed with the aim of reducing or eliminating the invasiveness caused by biopsy. The origins of the genetic materials tested by these methods remain unknown. In addition, these techniques lack diagnostic consistency with the reported genetic testing accuracy ranging from 30.4 to 97.8% [7, 8].

Morphological evaluation, a non-invasive method for blastocyst selection, is universally used in IVF clinics. Embryologists manually grade each blastocyst by visually observing its morphological characteristics [9, 10], including the degree of expansion and hatching status (1: less than half expansion, up to 6: fully hatched), cohesiveness and number of trophectoderm (TE) cells (A-C, from highest to lowest), and size and compactness of the inner cell mass (ICM; A-C, from highest to lowest). It has been reported that blastocysts with a lower grade in either TE cells or ICM have a lower euploidy rate [11–13]. Due to the qualitative grading criteria and inherent subjectivity, manual grading suffers from large intra/inter-observer variability [14, 15]. Morphokinetic features have also been used to evaluate embryo development patterns for embryo selection. Several morphokinetic parameters such as time to two cell stage (t2) [16] and time to full blastocyst (tB) [17, 18] showed significant delays in aneuploid embryos compared to euploid embryos. However, the ability of morphokinetic models to predict euploidy remains controversial, and significant disparities exist in the selection of morphokinetic events [19, 20].

Artificial intelligence (AI) has gained traction for non-invasive prediction of embryo ploidy status over the past five years. Various machine learning models that used different features as input have been investigated [21]. Manual morphological grading and morphokinetic annotation were most often used as the model input, where the euploidy prediction accuracy ranged from 64 to 72% and the area under the receiver operating characteristic curve (AUC) ranged from 0.67 to 0.75 [22–27]. Among these models, logistic regression and random forest achieved an AUC higher than 0.7 [23, 26, 27].

Instead of relying on manual grading and annotation, deep learning performs euploidy prediction by directly using images or time-lapse videos without involving manual evaluation. Image-based deep learning models achieved a euploidy accuracy of 69.3–77.4% and an AUC of 0.65 to 0.87 [28–31]. An ensemble model including DenseNet-161, ResNet-50 and DenseNet-121 sub-models achieved the highest AUC of 0.87 after data cleansing [29]. Video-based deep learning models achieved a euploidy accuracy of 71.4–73% and an AUC of 0.74 to 0.811 [32–36], where visual-temporal contrastive learning achieved the highest AUC of 0.811 [36].

Although these deep learning models eliminated human subjectivity and demonstrated improved performance of euploidy prediction, it is challenging to open the so-called ‘black box’ and understand the clinical aspects underlying these complex neural networks. The limited interpretability raises epistemic and ethical concerns, impeding the application of these models in clinical IVF [21, 37]. In addition, existing methods were all based on 2D images or videos (i.e., a single 2D image, 2D images from different focal planes, or a series of 2D images from time-lapse videos), which only included partial TE and ICM information. For the same blastocyst, evaluation results can vary with different blastocyst orientations [38].

This study aims to develop a new approach for non-invasive euploidy prediction in human blastocysts. In our approach, the morphological parameters of TE cells and ICM are first quantified via 3D morphology measurement and then used as the input of the prediction model. The prediction performance of six machine learning models was compared, and via model interpretation, quantitative criteria were generated for euploidy prediction with a prediction accuracy of 95.6%.

## Methods

### Study design and participants

This retrospective cohort study was performed at the CReATe Fertility Centre in Toronto, Canada from February 2022 to May 2023. Data were obtained from 226 unhatched blastocysts from 55 patients. Multi-view images of each blastocyst were collected on Day 6 (136–142 h after insemination). PGT-A results were used as the ground truth outcome. The study was non-interventional, and results were not used to make treatment decisions. The data analysis was conducted following the research protocol approved by Veritas independent institutional review board (#2022-2602-9773-1). Informed consent was not necessary for participation in this study since all data were retrospectively collected and fully de-identified with no intervention in embryo fate or patient care.

### Image capture

Multi-view images were captured by rotating the blastocyst during the preparation stage of TE biopsy. An embryologist held the blastocyst using a holding micropipette. The focal plane was first placed in the middle plane of the blastocyst to capture the first image. This step is only conducted once for each blastocyst. The focal plane was then moved downwards until individual TE cells and ICM were clearly visible. This focal plane was then fixed for image capturing throughout subsequent blastocyst rotations. A biopsy micropipette was used to gently push the blastocyst and rotate the blastocyst each time by a small angle, for instance, smaller than 35° such that more than 10 images were captured for the entire 360° rotation to achieve high-accuracy measurement. The rotation angle did not need to be precisely controlled as long as there was an overlap between two adjacent images. An image of the blastocyst was captured at each rotation. Alternatively, to avoid interruption by frequently capturing images, the entire rotation process was video recoded, and multi-view images of each blastocyst were exacted from the video afterwards. Supplementary Video 1 shows details of the blastocyst rotation process.

### 3D morphology measurement

**Fig. 1:**
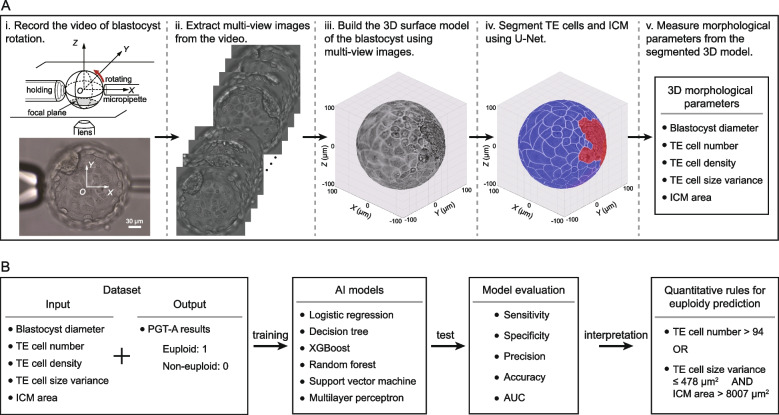
Schematic of non-invasive euploidy prediction in human blastocysts: **A** 3D morphology measurement. Multi-view images were captured during blastocyst rotation. A 3D blastocyst model was then built by projecting multi-view images to the spherical surface via transformation matrices calculated by SR-SIFT. Using U-Net, TE cells and ICM on the 3D surface model were segmented and 3D morphological parameters were measured. **B** Overview of the machine learning model development for euploidy prediction. In training, the input was the five morphological parameters quantified via 3D morphology measurement, and the output was the PGT-A results as the ground truth outcome. All six machine learning models were trained using the same training dataset. An additional test dataset was used to evaluate the performance of the models. Interpretation was conducted on the best-performing model where quantitative rules were generated for euploidy prediction

To quantify the morphology of a blastocyst three-dimensionally, the center *O* and diameter *D* of the blastocyst were measured from the image captured in the middle plane of the blastocyst and used to construct a spherical surface Ω. As shown in Fig. 1A, all multi-view images of the blastocyst were then cropped into *D*×*D* centered at *O*. Spherical rotation SIFT (SR-SIFT) algorithm was performed among multi-view images to calculate their transformation matrices [38], based on which multi-view images were projected on the spherical surface Ω to form the 3D surface model of the blastocyst. After 3D modeling, the TE cells and ICM of the blastocyst were segmented using U-Net, and their morphological parameters were measured from the segmented 3D surface model. The measurement error of the morphological parameters was less than 6.7%. More technical details can be found in our previous work [38] and Supplementary Video 2.

According to the current morphological grading system [9, 10], the morphological parameters quantified in this study were blastocyst diameter, TE cell number, TE cell density, TE cell size variance, and ICM area. Blastocyst diameter was used to quantify the blastocyst size. TE cell number, density and size variance were used to quantify TE cell morphological properties, where TE cell density was defined as TE cell number per 1,000 µm^2^ and TE cell size variance is defined as the standard deviation of the areas of all TE cells within a blastocyst. ICM area was used to denote ICM size.

### TE biopsy and PGT-A

TE biopsy was performed immediately after blastocyst rotation. Laser pulses were used to separate 4–5 TE cells from the blastocyst. Biopsied samples were amplified and analyzed by next-generation sequencing (NGS, Illumina) at the CReATe Fertility Centre. Blastocysts were classified as euploid, mosaic, and aneuploid that corresponded to < 20%, 20–80%, and > 80% aneuploidy, respectively.

### Machine learning models

All five parameters determined by 3D morphology measurement were used as the input of the machine learning models for euploidy prediction. The model output contains two categories: euploid and non-euploid (including mosaic and aneuploid). Figure 1B shows the development process of the prediction system. The prediction performance of six machine learning models, including logistic regression (LR), decision tree (DT), extreme gradient boosting (XGBoost), random forest (RF), support vector machine (SVM), and multilayer perceptron (MLP) commonly used in clinical studies were investigated.

The dataset was divided into 80% for training and validation, and 20% for test. The test dataset was not accessible to the machine learning models in the training and validation process. All hyperparameters of the machine learning models were tuned by k-fold cross-validation [39], and all models were trained to maximize their AUCs. For each model, the threshold corresponding to the point on the receiver operating characteristic (ROC) curve closest to the top-left point (0,1) was selected to maximize the sum of the model’s sensitivity and specificity [40]. Details for machine learning models are provided in Supplementary Table 1, and source codes are available at https://github.com/AMNL-UofT/euploidy-prediction.

### Statistical analysis

Statistical analysis was performed using IBM SPSS Statistics 26 and scikit-learn version 1.2.2 in Google Colaboratory. Categorical variables were described by number and percentage, and numerical variables were described by mean, standard deviation (SD) and range. The Chi-squared test was performed to analyze trends in categorical variables, and the t-test was performed to compare numerical variables among different groups. Pearson correlation was used to analyze the linear relationship among numerical variables. All statistical tests were two-tailed. *P* values of < 0.05 were considered statistically significant, and odd ratios (ORs) with 95% confidence interval (CI) were calculated. Forward stepwise logistic regression was used for multivariate analysis to calculate the adjusted odd ratios (aORs).

The performance of the machine learning models for euploidy prediction on the test dataset was evaluated by AUC, accuracy, precision, sensitivity, and specificity with 95% confidence interval. ROC curves among machine learning models were compared using DeLong’s test, and accuracy was compared by McNemar test. Feature importance was calculated to evaluate the predictive power of each feature on euploidy prediction. Details regarding evaluation metrics and feature importance are provided in Supplementary Table 1.

## Results

### Dataset

A total of 226 Day 6 blastocysts from 55 patients were used to train, validate, and test the machine learning models. The maternal age of the patients ranged from 21 to 44 years (34.4 ± 5.2 years). According to the PGT-A results, 57.1% (129/226) of the blastocysts were euploid, and 42.9% (97/226) were non-euploid, including mosaic (12.4%, 28/226) and aneuploid (30.5%, 69/226). The characteristics of the dataset are given in Supplementary Table 2. Supplementary Table 3 shows the euploidy rate of blastocysts assigned with different morphological grades. Overall, the euploidy rate decreased as the grades decreased from AA to CC in different maternal age groups. For blastocysts with grades ≥ BB, the euploidy rate decreased from 77.3 to 44.8% as maternal age increased.

The morphological parameters of all 226 blastocysts were successfully quantified via 3D morphology measurement. The pixel-to-micron scale of the parameters was calibrated by a standard calibration slide. Outliers were revisited by manually measuring the morphological parameters from the 3D surface model, and the outlier was corrected if its measurement error was larger than 8%. After preprocessing, the morphological parameters were then used as the input of the machine learning models for euploidy prediction. Among the 226 blastocysts, 181 were used for training and validation, and 45 were used for test. In the test dataset, 57.8% of the blastocysts were euploid and 42.2% non-euploid. All six models were tested under the same test dataset which was not used during model training and validation. The dataset of the morphological parameters and the corresponding PGT-A results is provided in Supplementary Table 5.

### Univariate analysis

Univariate analysis demonstrated that all five morphological parameters were significantly different between the euploid and non-euploid blastocysts (Table 1). The euploid blastocysts showed significantly larger diameters and ICM area, higher TE cell number and density, and lower TE cell size variance.

**Table 1:** Univariate and multivariate analysis of associations between 3D morphological parameters and ploidy status

Morphological parameters	All *n* = 226	Euploid *n* = 129	Non-euploid *n* = 97	Univariate analysis		Multivariate analysis	
				*P* value	OR (95% CI)	*P* value	aOR (95% CI)
Diameter (µm) (SD)	183.9 (21.6)	190.4 (19.1)	175.2 (21.9)	< 0.001	1.040 (1.023–1.056)	0.945	1.000
TE cell number (SD)	110.0 (49.7)	141.3 (38.5)	68.3 (27.1)	< 0.001	1.070 (1.052–1.089)	< 0.001	1.054 (1.034–1.073)
TE cell density (cell number /1000 µm^2^) (SD)	1.1 (0.4)	1.3 (0.3)	0.8 (0.3)	< 0.001	354.412 (80.111-1567.923	0.872	1.000
TE cell size variance (µm^2^) (SD)	467.6 (680.8)	221.5 (119.2)	794.9 (937.0)	< 0.001	0.992 (0.989–0.994	0.003	0.994 (0.991–0.998)
ICM area (µm^2^) (SD)	5492.7 (2280.7)	5883.4 (2303.1)	4973.2 (2154.3)	0.004	1.002 (1.001–1.003)	0.010	1.003 (1.001–1.006)

The blastocysts were further divided into three maternal age groups: ≤34 years in group A, 35–37 years in group B, and ≥ 38 years in group C. The blastocysts in group A showed a significantly smaller TE cell size variance than those in group B (386.4 µm^2^ vs. 582.7 µm^2^, *P* = 0.042). They also showed a higher TE cell number and density (117.1 vs. 95.9, *P* = 0.011; 1.1 cells/1000 µm^2^ vs. 1.0 cell/1000 µm^2^, *P* = 0.013), a smaller TE cell size variance (386.4 µm^2^ vs. 572.4 µm^2^, *P* = 0.033), and a higher euploidy rate (66.2% vs. 37.5%, *P* = 0.001) than the blastocysts in group C. However, among the euploid blastocysts, no statistically significant difference was found for any of the five morphological parameters among the three age groups. The same phenomenon was also observed among the non-euploid blastocysts. Table 2 shows the detailed distribution of the morphological parameters in the different age groups.

### Multivariate analysis

Multivariate analysis revealed that TE cell number, TE cell size variance and ICM area maintained statistical significance between the euploid and the non-euploid blastocysts (*P* < 0.001, aOR = 1.050, 95% CI 1.032–1.068; *P* = 0.002, aOR = 0.994, 95% CI 0.991–0.998; *P* = 0.005, aOR = 1.004, 95% CI 1.001–1.006), as shown in Table 1. Blastocyst diameter and TE cell density were not significant parameters associated with the ploidy status (*P* = 0.934; *P* = 0.837) compared to the other three parameters.

**Table 2:** Distribution of 3D morphological parameters in different maternal age groups

All blastocysts								
Morphological parameters	Maternal age				*P* value			
	Group A ≤ 34 *n* = 130	Group B 35–37 *n* = 48	Group C ≥ 38 *n* = 48		A vs. B		A vs. C	B vs. C
Diameter (µm) (SD)	185.7 (19.6)	181.1 (29.6)	181.8 (16.8)		0.325		0.222	0.893
TE cell number (SD)	117.1 (46.1)	104.8 (59.1)	95.9 (46.6)		0.267		0.011	0.187
TE cell density (cell number /1000 µm^2^) (SD)	1.1 (0.4)	1.1 (0.5)	1.0 (0.4)		0.653		0.013	0.162
TE cell size variance (µm^2^) (SD)	386.4 (525.2)	582.7 (880.4)	572.4 (802.5)		0.042		0.033	0.952
ICM area (µm^2^) (SD)	5590.0 (1995.3)	5737.0 (2907.4)	4985.1 (2273.7)		0.747		0.086	0.161
Euploidy rate (%)	66.2%	52.1%	37.5%		0.086		0.001	0.154
**Euploid blastocysts**								
**Morphological parameters**	**Maternal age**				***P*value**			
	**Group A** **≤ 34** *n* = 86	**Group B** **35–37** *n* = 25	**Group C** **≥ 38** *n* = 18		**A vs. B**		**A vs. C**	**B vs. C**
Diameter (µm) (SD)	190.2 (17.3)	191.6 (27.9)	189.6 (11.5)		0.756		0.888	0.745
TE cell number (SD)	142.2 (36.9)	139.6 (44.1)	139.4 (38.0)		0.440		0.501	0.913
TE cell density (cell number /1000 µm^2^) (SD)	1.3 (0.3)	1.3 (0.4)	1.3 (0.3)		0.868		0.944	0.850
TE cell size variance (µm^2^) (SD)	222.9 (112.1)	233.6 (156.5)	197.7 (93.4)		0.703		0.374	0.390
ICM area (µm^2^) (SD)	5795.5 (1828.3)	6418.9.0 (3342.6)	5559.1 (2622.3)		0.379		0.646	0.369

Feature importance was analyzed in all six machine learning models. TE cell number showed the highest importance in all models, followed by TE cell size variance and ICM area in LR, DT, SVM and MLP. ICM area was replaced by TE cell density in XGBoost and RF in the top-three ranked features. Feature rankings are summarized in Supplementary Fig. 1.

### Euploidy prediction results

**Fig. 2:**
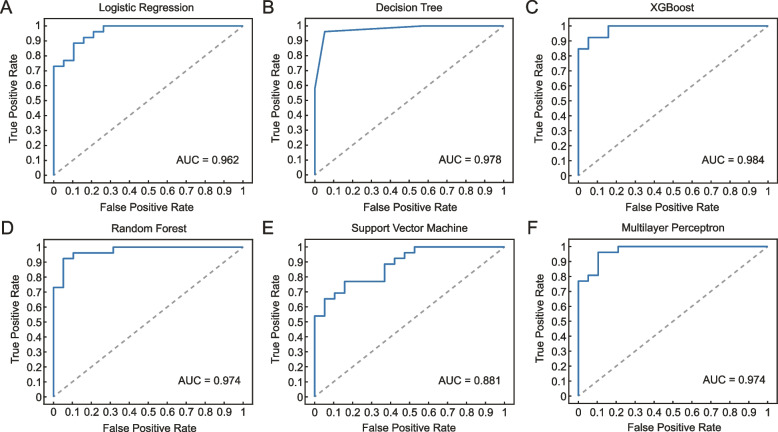
ROC curves of (**A**) logistic regression, (**B**) decision tree, (**C**) XGBoost, (**D**) random forest, (**E**) support vector machine, and (**F**) multilayer perceptron for predicting euploid blastocysts in the test dataset

With the five morphological parameters as input, the accuracy of euploidy prediction by the six models ranged from 80 to 95.6%, and the AUCs ranged from 0.881 to 0.984 (Fig. 2). The best-performing models for euploidy prediction were tree-based models, i.e., DT and XGBoost. DT achieved the highest accuracy of 95.6% (95% CI 84.9-99.5%) with the AUC of 0.978 (95% CI 0.882–0.999). The sensitivity, specificity and precision of DT were 96.2% (95% CI 80.4-99.9%), 94.7% (95% CI 74.0-99.9%), and 96.2% (95 CI 78.7-99.4%), indicating its strong ability to avoid either false positive or false negative results. XGBoost achieved the highest AUC of 0.984 (95% CI 0.892-1.000) with the accuracy of 93.3% (95% CI 81.7-98.6%). The pairwise comparison of ROC curves showed no significant difference between the AUCs of the DT model and the XGBoost model (*P* = 0.574). Performance metrics of all the machine learning models are described in Table 3.

The test dataset was further divided into different maternal age groups. Post-hoc analysis was conducted to investigate whether differences exist in the DT and XGBoost models for euploidy prediction on the basis of age. Details of prediction accuracy and AUCs are summarized in Supplementary Table 4. No significant difference was found among the age groups of ≤ 34, 35–37, and ≥ 38 years using either DT or XGBoost to predict euploid blastocysts.

Interpretation was conducted on the DT model. The rule used at each node of the tree was extracted from the model. Quantitative rules used by the DT model for euploidy prediction are summarized as follows: (1) if the TE cell number of a blastocyst on Day 6 is higher than 94, then the blastocyst is predicted to be euploid; or (2) if the TE cell size variance of the blastocyst is no larger than 478 µm^2^, and in the meantime its ICM area is larger than 8007 µm^2^, then the blastocyst is predicted to be euploid.

**Table 3:** Performance of all six machine learning models for euploidy prediction

Metrics	LR	DT	XGBoost
Sensitivity (95% CI)	88.5% (69.9-97.6%)	96.2% (80.4-99.9%)	92.3% (74.9-99.1%)
Specificity (95% CI)	89.5% (66.9-98.7%)	94.7% (74.0-99.9%)	94.7% (74.0-99.9%)
Precision (95% CI)	92.0% (75.5-97.7%)	96.2% (78.7-99.4%)	96.0% (78.0-99.4%)
Accuracy (95% CI)	88.9% (76.0-96.3%)	95.6% (84.9-99.5%)	93.3% (81.7-98.6%)
AUC (95% CI)	0.962 (0.854–0.996)	0.978 (0.882–0.999)	0.984 (0.892-1.000)
**Metrics**	**RF**	**SVM**	**MLP**
Sensitivity (95% CI)	92.3% (74.9-99.1%)	76.9% (56.4-91.0%)	96.2% (80.4-99.9%)
Specificity (95% CI)	94.7% (74.0-99.9%)	84.2% (60.4-96.6%)	84.2% (60.4-96.6%)
Precision (95% CI)	96.0% (78.0-99.4%)	87.0% (69.8-95.1%)	89.3% (74.6-95.9%)
Accuracy (95% CI)	93.3% (81.7-98.6%)	80.0% (65.4-90.4%)	91.1% (78.8-97.5%)
AUC (95% CI)	0.974 (0.876–0.999)	0.881 (0.749–0.958)	0.974 (0.876–0.999)

## Discussion

In this study, we conducted 3D quantitative morphological measurement of Day 6 blastocysts, based on which, machine learning models were used to non-invasively predict the euploid blastocysts. Five morphological parameters of TE cells and ICM were quantified, the results of which were used to train six machine learning models commonly investigated in clinical studies. The results showed that the tree-based models achieved the highest accuracy (95.6%) and AUC (0.984). To our best knowledge, these are the highest accuracy and AUC values reported in the literature for euploidy prediction.

Previous studies demonstrated that blastocysts with grade-A TE and ICM showed a significantly higher euploidy rate compared to grade-B or grade-C groups [12, 13]. For euploidy prediction with machine learning models, the gradings of TE and ICM were converted into numerical scores as the model input. LR and XGBoost were reported to give an accuracy of 63.2% [30] and an average AUC of 0.7 [22, 27]. Since manual grading was used as the model input, the prediction accuracy in these methods was subject to observer variances. For morphological grading, it was reported that the inter-observer agreement was fair (kappa = 0.349 for ICM grading; kappa = 0.397 for TE grading) [41] and the overall intra-observer agreement was moderate (kappa = 0.495) [42]. The observer variances introduce noises into the models, resulting in poor prediction consistency and limited prediction performance.

To eliminate human subjectivity, deep learning models directly using blastocyst images as the input were developed for euploidy prediction. Chavez-Badiola et al. [28] used a convolutional neural network (CNN) to exact features from a static image of a blastocyst and a fully connected network to generate the probability of euploidy. The model achieved an AUC of 0.74. Diakiw et al. [29] reported an ensemble model that included three CNN-based deep learning networks and used a majority-mean-based voting strategy for euploidy prediction. The ensemble model improved the AUC to 0.87 after data cleansing. Although these models eliminated human subjectivity, their prediction was based on 2D images that lack complete morphological information. Not all TE cells are visible in 2D images due to the spherical structure of blastocysts, and the size of the ICM appears different at different blastocyst orientations. Morphokinetic and clinical features have also been used as additional input to achieve an AUC up to 0.879 [27, 30, 33], which was a minor improvement over 0.87. A potential reason for the minor improvement was that the redundant information contained in morphokinetic or clinical features could have caused overfitting and thus limited the predictive ability of these models [21, 23, 30, 43].

In the present study, we quantified the morphological parameters of entire blastocysts using 3D morphology measurement. All five parameters showed significant differences between euploid and non-euploid blastocysts. Using them as input, DT, XGBoost, RF and MLP achieved > 0.9 in both accuracy and AUC. The high euploidy prediction accuracy was attributed to the strong associations between the 3D morphological parameters and ploidy status of the blastocyst, as well as the elimination of noises caused by input subjectivity and information loss. Notably, the lower bounds of 95% CI for the AUCs of DT and XGBoost were 0.882 and 0.892, higher than the AUCs of existing deep learning models for euploidy prediction. The higher lower bounds suggest that our models could maintain their outstanding performance as the dataset is further enlarged.

The parameter distribution was further investigated between different age groups. It was found that although the parameters and the euploidy rate among all the blastocysts showed significant differences in terms of the maternal age, the distribution difference of all five morphological parameters among euploid blastocysts was not significant between different age groups. The same phenomenon was also observed among non-euploid blastocysts. These results suggest that euploid blastocysts from different age groups conform to similar morphological characteristics. It also explains why our machine learning models were able to achieve high accuracies in different age groups although maternal age was not included in the model input.

In all six models, TE-related parameters (TE cell number and TE cell size variance) showed higher predictive abilities than the ICM-related parameter (ICM area), which is consistent with previously reported studies [13, 22, 44]. Since biopsy and PGT-A are performed on TE cells, the testing result is not necessarily representative of the ploidy status of the ICM. It has been reported that the concordance of ploidy status between TE and ICM ranged from 85 to 95% [45–47]. This disconcordance lowers the predictive power of the ICM area on the PGT-A outcome. Additionally, the TE layer shows distinct morphological details of individual cells due to the monolayer structure compared to the ICM which is a compact, fist-like structure with no visible details of individual cells. Thus, the models tended to assign higher weights to TE-related features since the models were able to acquire more information from them than from the ICM.

One controversial issue of machine learning models used for medical treatment is their interpretability. Although current deep learning models have demonstrated AUCs higher than 0.8 for euploidy prediction [27, 29, 33], it is difficult to understand clinical aspects underlying these complex neural networks. The limited interpretability raises concerns over whether the decisions made by a poorly understood model can be trusted for IVF treatment [21, 37]. In our study, we conducted model interpretation on the DT model which achieved the highest accuracy of 95.6%. The high euploidy prediction accuracy was attributed to the strong associations between the 3D morphological parameters and ploidy status of the blastocyst as well as the elimination of noises caused by input subjectivity and information loss. The quantitative criteria extracted from the DT model showed that the majority of the euploid blastocysts (120 out of 129 euploid blastocysts) had a high number of TE cells (> 94). The minority of the euploid blastocysts (3 out of 129 euploid blastocysts) had a lower number of TE cells (72–78) but a large ICM (> 8,007 µm^2^) and their TE cells showed a small variation in size (≤ 478 µm^2^).

These findings could be understood from previous studies in human blastocysts. Evidence suggested that mosaic and aneuploid human blastocysts have a significantly lower number of TE cells and a smaller ICM size due to the increased apoptosis rate and reduced proliferation rate [48–51]. In both TE and ICM, chromosomal imbalance causes significant differences in gene expression. The differential gene expression causes the disruption of the pathways involved in the regulation of apoptosis and proliferation [48, 50, 51]. Specifically, Regin et al. [50] found that the abnormal gene expression disrupted the intracellular protein homeostasis and caused proteotoxic stress. Autophagy was then activated to mitigate the stress. Unsuccessful recovery of the protein homeostasis by autophagy further triggered the apoptosis process to eliminate the cell. The increased apoptosis rate was found to be more pronounced among aneuploid cells in the TE layer compared to the ICM [50, 51], which may explain the higher predictive power of TE-related morphological parameters compared to the ICM-related parameter in our machine learning model. Furthermore, the proteotoxic stress can also cause DNA damage during DNA replication in mitosis [49, 52]. In human blastocysts, such DNA damage leads to cell-cycle arrest, and thereby delay of proliferation [50, 51]. The increased apoptosis and reduced proliferation collectively result in a lower number of TE cells and a smaller ICM size in mosaic and aneuploid blastocysts than in euploid blastocysts.

Regarding the TE cell size variances, studies in human cells suggested that polyploid cells have larger cell sizes than euploid cells. Williams et al. [53] found that the size of trisomic cells in human fibroblasts was significantly larger than that of euploid cells, as a result of abnormal cellular metabolism (e.g., increased glutamine consumption). Neurohr et al. [54] also found that human fibroblasts with tetraploidy or greater ploidy increased in cell size when they entered cell-cycle arrest due to DNA damage during mitosis. These findings indicate that non-euploid blastocysts containing both polyploid cells and euploid cells in the TE layer may possess a larger cell size variance than euploid blastocysts.

A few limitations exist in the present study and deserve further investigation. Firstly, this study did not include data from Day 5 embryos. All biopsies were conducted on Day 6 blastocysts (136–142 h after insemination) since a high percentage of Day 5 embryos were still at an early stage of blastocysts [55]. The correlation between 3D morphological parameters and ploidy status of Day 5 blastocysts needs further investigation. Secondly, this study aims to differentiate between euploid blastocysts and non-euploid ones. Mosaic embryos have a mixture of euploid and aneuploid cells. The overall incidence of mosaicism at the blastocyst stage ranges from 5 to 15% [56, 57]. Due to the low rate of mosaicism (12.4%), in this study, mosaic and aneuploid blastocysts were assigned to the same category of non-euploid. Compared to euploid embryos, mosaic embryos tend to have lower reproductive potential [58]. Among mosaic embryos, low-level and segmental mosaic embryos have a higher live birth rate than high-level and whole-chromosome mosaic embryos [59]. With a larger dataset, non-euploid would be further categorized into different mosaic and aneuploid categories as shown in Supplementary Table 2. Thirdly, this study was based on data collected from a single clinical center. One focus in our ongoing work is to collect a larger dataset from multiple centers, subanalyze the specific types of aneuploid and mosaic embryos, and further evaluate the performance of ploidy prediction from 3D morphological parameters. Furthermore, a prospective blinded study is needed to evaluate the prediction accuracy using this technology.

## Conclusions

This retrospective study showed strong predictive abilities of morphological parameters obtained by 3D morphological measurement for euploidy prediction in human blastocysts. The proposed system eliminated the input subjectivity from observers and information loss caused by the usage of 2D images. Using the five 3D morphological parameters, DT achieved the highest accuracy of 95.6% and XGBoost achieved the highest AUC of 0.984 among all six machine learning models. The prediction performance maintained among different age groups. Model interpretation was conducted and quantitative criteria were extracted from the DT model. Our prediction system may facilitate the comprehension of associations between blastocysts’ morphology and their ploidy status, provide a standardized and non-invasive method for morphological evaluation of human blastocysts, and aid decision-making for embryo selection in IVF treatments.

## Supplementary Information

Supplementary Material 1.

Supplementary Material 2.

Supplementary Material 3.

## Data Availability

The morphological parameters of blastocysts obtained by 3D morphology measurement and their corresponding PGT-A results used in this study are provided in Supplementary Table 5. Source codes of machine learning models developed in this study are available at https://github.com/AMNL-UofT/euploidy-prediction. The blastocyst videos and patient data underlying this article will be shared on reasonable request to the corresponding author.

## Abbreviations

AI: Artificial intelligence
aOR: Adjusted odd ratio
AUC: Area under the receiver operating characteristic curve
BFS: Blastocoel fluid sampling
CI: Confidence interval
CNN: Convolutional neural network
ICM: Inner cell mass
IVF: *in vitro* fertilization
LR: Logistic regression
MLP: Multilayer perceptron
NGS: Next-generation sequencing
OR: Odd ratio
PGT-A: Preimplantation genetic testing for aneuploidy
RF: Random forest
ROC: Receiver operating characteristic
SBM: Spent blastocyst media
SD: Standard deviation
SR-SIFT: Spherical rotation SIFT
SVM: Support vector machine
t2: Time to two cell stage
tB: Time to full blastocyst
TE: Trophectoderm
XGBoost: Extreme gradient boosting
