# Supplementary material for: Non-invasively predicting euploidy in human blastocysts via quantitative 3D morphology measurement: a retrospective cohort study
Source: Reprod Biol Endocrinol. 2024 Oct 28;22:132. doi: 10.1186/s12958-024-01302-x (PMC11514912; doi:10.1186/s12958-024-01302-x)
Supplement: Supplementary file 1 — Supplementary Material 1. [file 12958_2024_1302_MOESM1_ESM.docx]

**Supplementary Material**

**Supplementary Fig. 1** Feature importance of (**A**) logistic regression, (**B**) decision tree, (**C**) XGBoost, (**D**) random forest, (**E**) support vector machine, and (**F**) multilayer perceptron using 3D morphological parameters as the input for predicting euploidy in human blastocysts.

**Supplementary Table 1** Terminology definitions of machine learning modeling and evaluation

**Supplementary Table 2** Characteristics of dataset used to develop machine learning models for euploidy prediction in human blastocysts

**Supplementary Table 3** Euploidy rate of different morphological grades in terms of different maternal age groups

**Supplementary Table 4** Performance of decision tree and XGBoost for euploidy prediction in terms of different maternal age groups

**Supplementary Table 5** Morphological parameters of 226 human blastocysts quantified by 3D morphology measurement and their corresponding PGT-A results

**Supplementary Video 1** Details of the blastocyst rotation process

**Supplementary Video 2** Technical details of 3D morphology measurement for quantifying 3D morphological parameters of human blastocysts

**
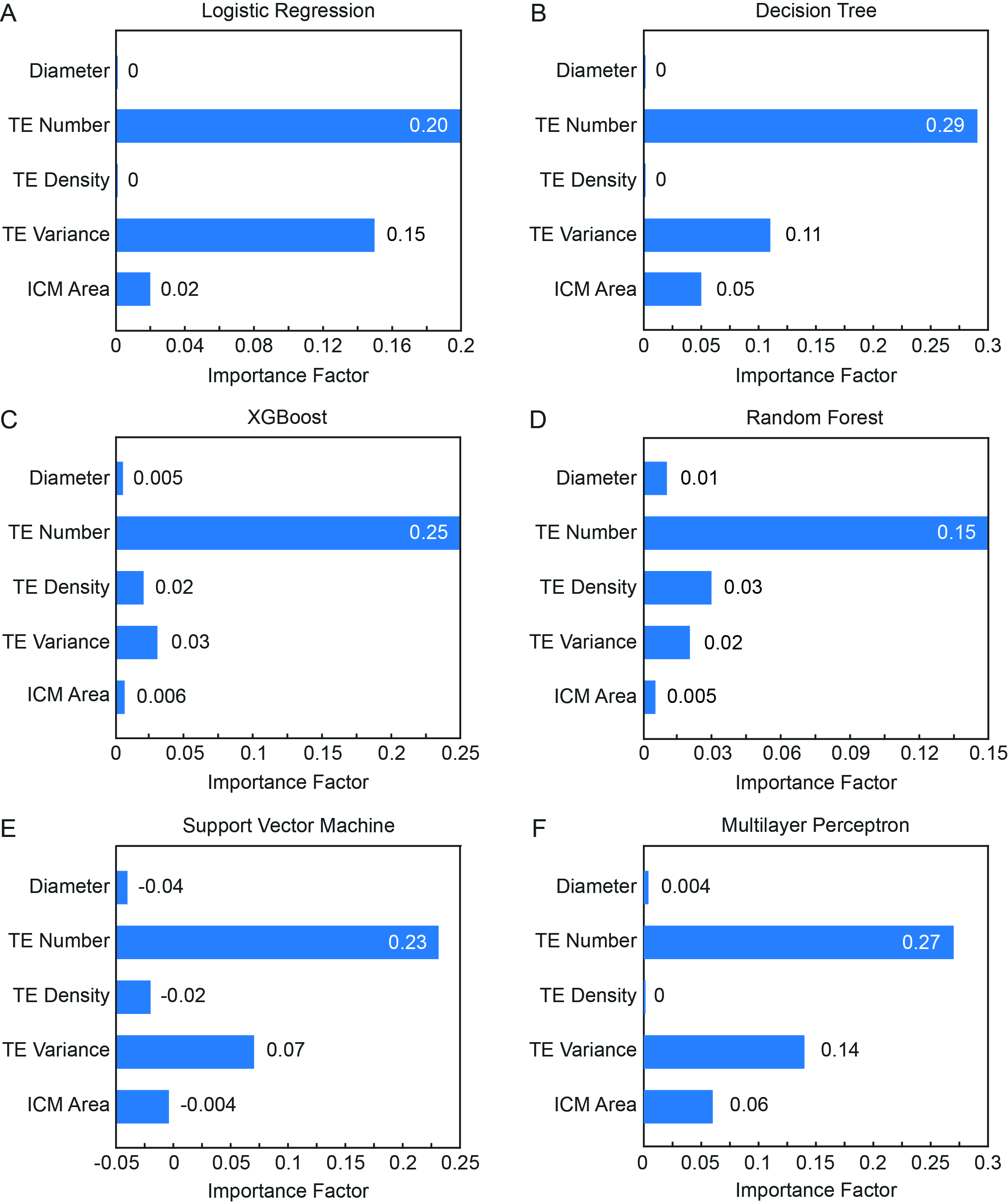
**

**Supplementary Fig. 1** Feature importance of (**A**) logistic regression, (**B**) decision tree, (**C**) XGBoost, (**D**) random forest, (**E**) support vector machine, and (**F**) multilayer perceptron using 3D morphological parameters as the input for predicting euploidy in human blastocysts.

**Supplementary Table 1** Terminology definitions of machine learning modeling and evaluation

| **Term** | **Definition** |
| --- | --- |
| Logistic regression (LR) | LR uses the logistic function 1/(1+*e*^-^***^βX^***) to describe the relationship between the input variables ***X*** and the dependent variable. It is a generalized linear model which uses the linear combination ***βX*** of all independent variables for modeling. This relatively simple function has high interpretability but limits its application to complex scenarios. For LR training in this study, multivariate analysis was first conducted among all morphological parameters. Parameters with a *P*-value of <0.15 for euploidy prediction were selected as the input for LR modeling. For LR training, the coordinate descent algorithm was used for model optimization, and L1 regularization was used in the cost function to prevent overfitting. |
| Decision tree (DT) | DT is a flowchart-like tree structure that applies a series of rules at the tree nodes to repeatedly split the dataset into different subgroups until no statistical significance is produced for further division. Subgroups at the terminal nodes obtain their classification results. Since DT is a rule-based method rather than a ‘black box’, it is easy to translate the final model to a set of rules. The interpretability enables the understanding of its logic in terms of clinical aspects and facilitates its application in medicine. For DT training in this study, entropy was used to find the optimal splitting at each node. To avoid overfitting, the maximum depth of tree was set to be 3, and the minimal cost-complexity pruning was used to reduce the tree complexity. |
| Extreme gradient boosting (XGBoost) | XGBoost, an ensemble learning model based on the decision tree, produces a group of trees for prediction. During the training process, these trees are added sequentially with new trees aiming to reduce the errors of the prior trees. For XGBoost training in this study, a total of 200 trees were produced and fast histogram-based approximate greedy was used for tree construction. |
| Random forest (RF) | RF, another ensemble learning model based on the decision tree, produces a group of independent trees for prediction. Final prediction result is determined by the majority votes from all trees. For RF training in this study, a total of 500 trees were produced, and bootstrap aggregating (also called bagging) was used to enhance tree diversity and avoid overfitting. |
| Support vector machine (SVM) | SVM projects each data point in the dataset from the original feature space to a higher dimensional space using linear or nonlinear kernel functions. In the new dimensional space, a hyperplane is constructed to classify the dataset into two classes. After increasing the dimensions, it becomes difficult to explain the meaning of a data point in the new dimensional space, resulting in poor interpretability. For SVM training in this study, linear kernels were selected and L2 regularization was used in the cost function to avoid overfitting. |
| Multilayer perceptron (MLP) | MLP is a deep learning neural network with an input layer, an output layer and multiple hidden layers. It is able to describe the nonlinear relationship between the input parameters and the output classification results by using nonlinear activation functions. It is considered as a ‘black box’ model since the complex neural network structures limit its interpretability. For MLP training in this study, logistic sigmoid function was used as the activation function. Stochastic gradient-based optimization was used to find optimal model parameters, and L2 regularization was used in the cost function to avoid overfitting. |
| True positive (TP) | A true positive prediction is an outcome where a euploid blastocyst is correctly predicted as euploidy. |
| True negative (TN) | A true negative prediction is an outcome where an aneuploid blastocyst is correctly predicted as aneuploidy. |
| False positive (FP) | A false positive prediction is an outcome where an aneuploid blastocyst is incorrectly predicted as euploidy. |
| False negative (FN) | A false negative prediction is an outcome where a euploid blastocyst is incorrectly predicted as aneuploidy. |
| Sensitivity | Sensitivity is also called true positive rate (TPR) or recall. High sensitivity indicates high true positive and low false negative. It is described by  $Sensitivity=\frac{\mathrm{TP}}{TP+FN}$ |
| Specificity | Specificity is also called true negative rate (TNR). High specificity indicates high true negative and low false positive. It is described by  $Specificity=\frac{\mathrm{TN}}{TN+FP}$ |
| Precision | Precision is also called positive predictive value (PPV). High precision indicates high true positive and low false positive. It is described by  $Precision=\frac{\mathrm{TP}}{TP+FP}$ |
| Accuracy | Accuracy evaluates the number of correct predictions over the total number of predictions, which is described by  $Accuracy=\frac{TP+TN}{TP+TN+FP+FN}$ |
| ROC curve | The ROC curve is the plot of the true positive rate against the false positive rate at different threshold of classification. |
| AUC | AUC is defined as the area under the ROC curve, which evaluates the overall model performance regardless of classification threshold. |
| Threshold | The threshold is the value used to classify the output probability or scores into different categories. In this study, for each model, the threshold corresponding to the point on the ROC curve closest to the top-left point (0,1) was selected to maximize the sum of the model’s sensitivity and specificity. |
| Permutation feature importance | The permutation importance of a feature is defined to be the decrease of AUC when the value of this feature is randomly shuffled. A high permutation importance indicates a strong ability of the feature in euploidy prediction. Negative permutation importance means that the feature does not contribute to euploidy prediction. |

**Supplementary Table 2** Characteristics of dataset used to develop machine learning models for euploidy prediction in human blastocysts

| **Characteristics** | **N (%), Mean (SD)** |
| --- | --- |
| Total number of patients | 55 |
| Number of embryos per patient | 4.1 (2.9) |
| Maternal age (year) | 34.4 (5.2) |
| Total number of embryos | 226 |
| Embryo diameter (µm) | 183.9 (21.6) |
| ICM grade |  |
| A | 56 (24.8%) |
| B | 115 (50.9%) |
| C | 55 (24.3%) |
| TE grade |  |
| A | 45 (19.9%) |
| B | 115 (50.9%) |
| C | 66 (29.2%) |
| PGT-A results |  |
| Euploid | 129 (57.1%) |
| Mosaic |  |
| Segmental chromosome^a^ | 6 (2.7%) |
| Single whole chromosome^b^ | 14 (6.2%) |
| Complex chromosomes^c^ | 8 (3.5%) |
| Aneuploid |  |
| Segmental chromosome^a^ | 8 (3.5%) |
| Single whole chromosome^b^ | 28 (12.4%) |
| Complex chromosomes^c^ | 33 (14.6%) |

^a^ Mosaic/aneuploid embryos with exclusively segmental abnormalities.

^b^ Mosaic/aneuploid embryos with single whole-chromosome abnormalities.

^c^ Mosaic/aneuploid embryos with whole-chromosome abnormalities in more than one chromosome or combinations of whole-chromosome and segmental abnormalities.

**Supplementary Table 3** Euploidy rate of different morphological grades in terms of different maternal age groups

| **Morphological grade** | **Euploidy rate (%)** | | | |
| --- | --- | --- | --- | --- |
|  | **Group A**  **maternal age ≤34**  **n=130** | **Group B**  **maternal age 35-37**  **n=48** | **Group C**  **maternal age ≥38**  **n=48** | **All**  **n=226** |
| AA | 94.4% (17/18) | 100.0% (5/5) | 66.7% (2/3) | 92.3% (24/26) |
| AB | 76.9% (10/13) | 71.4% (7/9) | 50.0% (4/8) | 70% (21/30) |
| BA | 76.9% (10/13) | 66.7% (2/3) | 33.3% (1/3) | 68.4% (13/19) |
| BB | 70.5% (31/44) | 46.7% (7/15) | 40.0% (6/15) | 59.5% (44/74) |
| ≥BB | 77.3% (68/88) | 65.6% (21/32) | 44.8% (13/29) | 68.5% (102/149) |
| BC | 35.7% (5/14) | 33.3% (1/3) | 25.0% (1/4) | 33.3% (7/21) |
| CB | 62.5% (5/8) | 50.0% (1/2) | 0.0% (0/2) | 50.0% (6/12) |
| CC | 40.0% (8/20) | 18.2% (2/11) | 30.8% (4/13) | 31.8% (14/44) |

**Supplementary Table 4** Performance of decision tree and XGBoost for euploidy prediction in terms of different maternal age groups

| **Decision tree** | | | | | | | | |
| --- | --- | --- | --- | --- | --- | --- | --- | --- |
| **Metrics** | **Maternal age** | | |  | | ***P* value** | | |
|  | **Group A**  **≤34**  **n=21** | **Group B**  **35-37**  **n=11** | **Group C**  **≥38**  **n=13** | | **A vs. B** | | **A vs. C** | **B vs. C** |
| Accuracy (95% CI) | 100.0% (83.9%-100.0%) | 100.0% (71.5%-100.0%) | 84.6% (54.6%-98.1%) | | 1.000 | | 0.289 | 0.375 |
| AUC  (95% CI) | 1.000 (0.839-1.000) | 1.000 (0.715-1.000) | 0.925 (0.642-0.998) | | 1.000 | | 0.290 | 0.290 |
| **XGBoost** | | | | | | | | |
| **Metrics** | **Maternal age** | | |  | | ***P* value** | | |
|  | **Group A**  **≤34**  **n=21** | **Group B**  **35-37**  **n=11** | **Group C**  **≥38**  **n=13** | | **A vs. B** | | **A vs. C** | **B vs. C** |
| Accuracy (95% CI) | 95.2% (76.2%-99.9%) | 100.0% (71.5%-100.0%) | 84.6% (54.6%-98.1%) | | 1.000 | | 0.453 | 0.375 |
| AUC  (95% CI) | 1.000 (0.839-1.000) | 1.000 (0.715-1.000) | 0.950 (0.676-1.000) | | 1.000 | | 0.403 | 0.403 |

**Supplementary Table 5** Morphological parameters of 226 human blastocysts quantified by 3D morphology measurement and their corresponding PGT-A results

| **Diameter** | **TE number** | **TE density** | **TE size variance** | **ICM area** | **PGT-A** | **Details** |
| --- | --- | --- | --- | --- | --- | --- |
| 210.22 | 201 | 1.55 | 125.95 | 8837.95 | Euploid |  |
| 169.05 | 88 | 1.04 | 288.04 | 5027.40 | Aneuploid | +21; (+9, 30%); (+13, 30%) |
| 201.02 | 171 | 1.39 | 104.27 | 4085.73 | Euploid |  |
| 208.84 | 12 | 0.09 | 4268.27 | 5016.63 | Aneuploid | -7; -8 |
| 197.80 | 118 | 1.04 | 658.27 | 9602.11 | Euploid |  |
| 184.23 | 46 | 0.46 | 2251.71 | 5634.54 | Mosaic | (-2, 50%); (-4, 50%); (+11, 50%); (+17, 30%); (+X, 30%) |
| 205.84 | 42 | 0.34 | 3312.14 | 10408.92 | Aneuploid | -22 |
| 199.18 | 102 | 0.87 | 306.07 | 6977.62 | Euploid |  |
| 229.08 | 162 | 1.05 | 529.36 | 10353.54 | Euploid |  |
| 195.80 | 106 | 0.93 | 237.60 | 6372.70 | Euploid |  |
| 183.77 | 28 | 0.28 | 3139.32 | 7556.50 | Aneuploid | -8 |
| 186.99 | 136 | 1.41 | 395.35 | 13586.62 | Euploid |  |
| 273.01 | 162 | 0.73 | 391.93 | 13632.33 | Euploid |  |
| 199.64 | 159 | 1.41 | 511.91 | 12724.78 | Euploid |  |
| 195.50 | 55 | 0.48 | 1698.82 | 5610.52 | Aneuploid | -4 |
| 207.92 | 156 | 1.24 | 402.13 | 9661.55 | Euploid |  |
| 186.99 | 135 | 1.30 | 290.44 | 5742.51 | Euploid |  |
| 164.68 | 75 | 0.97 | 315.51 | 8117.51 | Euploid |  |
| 161.23 | 70 | 0.93 | 645.18 | 6704.49 | Aneuploid | -14 |
| 189.75 | 144 | 1.38 | 344.43 | 8857.84 | Euploid |  |
| 206.08 | 24 | 0.19 | 4466.67 | 5891.74 | Aneuploid | +14; -15 |
| 214.36 | 120 | 0.85 | 464.81 | 3769.81 | Euploid |  |
| 208.45 | 145 | 1.15 | 216.75 | 9924.53 | Euploid |  |
| 177.33 | 125 | 1.39 | 365.86 | 8834.20 | Euploid |  |
| 158.93 | 54 | 0.73 | 328.70 | 5417.33 | Euploid |  |
| 186.30 | 120 | 1.18 | 278.11 | 7373.04 | Euploid |  |
| 215.51 | 114 | 0.83 | 352.98 | 7753.76 | Euploid |  |
| 231.00 | 181 | 1.13 | 432.70 | 7936.03 | Euploid |  |
| 198.95 | 174 | 1.51 | 497.39 | 8823.50 | Euploid |  |
| 239.20 | 134 | 0.78 | 236.40 | 8423.50 | Euploid |  |
| 205.16 | 126 | 1.00 | 238.25 | 5947.92 | Euploid |  |
| 201.34 | 138 | 1.18 | 312.50 | 10023.49 | Euploid |  |
| 167.21 | 84 | 0.99 | 530.94 | 2910.98 | Aneuploid | +13q12.3q22.1; -21 |
| 178.94 | 139 | 1.45 | 258.79 | 4435.45 | Euploid |  |
| 212.29 | 45 | 0.33 | 2982.13 | 6666.35 | Aneuploid | +20q13.12q13.33 |
| 184.69 | 72 | 0.70 | 1163.05 | 4983.50 | Mosaic | (+11q22.3q25, 30%); (+13q32.3q34, 30%) |
| 166.75 | 78 | 1.00 | 469.36 | 9402.08 | Euploid |  |
| 192.28 | 102 | 0.97 | 520.40 | 10850.37 | Euploid |  |
| 174.80 | 39 | 0.43 | 1014.24 | 4727.83 | Aneuploid | -2q36.3q37.3; -6p25.3q22.31 |
| 201.71 | 120 | 0.97 | 219.44 | 4508.35 | Euploid |  |
| 181.47 | 69 | 0.72 | 1537.45 | 6983.91 | Mosaic | (+7, 45%) |
| 190.67 | 96 | 0.88 | 430.84 | 5136.70 | Euploid |  |
| 189.52 | 102 | 0.94 | 615.03 | 4254.54 | Euploid |  |
| 153.41 | 42 | 0.63 | 831.58 | 6835.16 | Mosaic | (-2p, 55%); (-22, 55%); (-13,30%) |
| 177.79 | 78 | 0.88 | 713.81 | 10493.30 | Mosaic | (+2, 30%) |
| 204.24 | 66 | 0.52 | 1192.37 | 5122.89 | Aneuploid | +3; +15 |
| 185.61 | 84 | 0.84 | 187.35 | 7896.81 | Aneuploid | +4q11q28.1; -4q28.1q35.2 |
| 168.59 | 63 | 0.73 | 266.16 | 3498.81 | Mosaic | (-4, 50%) |
| 202.86 | 126 | 1.06 | 330.34 | 9856.59 | Euploid |  |
| 171.12 | 153 | 1.73 | 118.53 | 3428.13 | Euploid |  |
| 166.06 | 162 | 1.94 | 139.32 | 3111.00 | Euploid |  |
| 177.79 | 179 | 1.87 | 125.44 | 3712.42 | Euploid |  |
| 186.99 | 88 | 0.82 | 448.39 | 3104.54 | Aneuploid | -15 |
| 184.23 | 181 | 1.77 | 151.56 | 4216.45 | Euploid |  |
| 191.36 | 62 | 0.58 | 966.05 | 8446.65 | Mosaic | (-15, 40%) |
| 203.09 | 111 | 0.94 | 288.77 | 12109.87 | Euploid |  |
| 190.21 | 114 | 1.05 | 175.62 | 4669.11 | Euploid |  |
| 158.47 | 26 | 0.34 | 1396.00 | 2328.08 | Mosaic | (+6q, 60%) |
| 135.93 | 30 | 0.60 | 971.26 | 7814.34 | Aneuploid | +10;(+15,70%); (-3, 60%); (-5, 60%); (-6, 30%); (-8, 30%); (+9, 40%); (+13, 40%); (-16, 40%); (-14, 50%); (-16, 60%); (-19, 40%); (+21, 35%); (+22, 45%) |
| 172.27 | 38 | 0.42 | 1631.69 | 3732.62 | Aneuploid | +13; -15 |
| 204.01 | 82 | 0.65 | 1254.87 | 4533.69 | Aneuploid | +14q23.3q32.3; +16; (-Y, 50%) |
| 187.45 | 40 | 0.39 | 1905.52 | 6570.39 | Aneuploid | +7; +21 |
| 150.65 | 6 | 0.10 | 5166.36 | 9096.37 | Aneuploid | -10q22.3q26.3; -22; (+12, 60%); (+6, 45%); (+19p,40%) |
| 172.87 | 32 | 0.40 | 1527.24 | 13579.25 | Aneuploid | -10q; (+10p, 30%) |
| 196.98 | 75 | 0.67 | 1147.32 | 9265.25 | Mosaic | (-21, 50%) |
| 173.88 | 58 | 0.61 | 392.62 | 0.00 | Aneuploid | +2; +9; +13; -14; +19; (+15, 60%) |
| 161.23 | 40 | 0.51 | 664.62 | 2607.44 | Aneuploid | +7; +22; (-2q21.1q37.3, 30%) |
| 181.24 | 126 | 1.31 | 260.53 | 7296.66 | Euploid |  |
| 174.57 | 117 | 1.33 | 222.15 | 7487.25 | Euploid |  |
| 193.43 | 92 | 0.82 | 241.45 | 5751.82 | Aneuploid | -7p22.3p11.2; +7q11.1q36.3 |
| 189.29 | 135 | 1.25 | 146.11 | 4704.34 | Euploid |  |
| 172.73 | 159 | 1.79 | 128.25 | 5125.16 | Euploid |  |
| 144.21 | 96 | 1.57 | 312.78 | 4249.19 | Mosaic | (-13q33.2q34, 50%) |
| 191.82 | 165 | 1.50 | 122.88 | 5584.76 | Euploid |  |
| 173.19 | 147 | 1.66 | 99.65 | 5532.07 | Euploid |  |
| 187.91 | 150 | 1.42 | 106.92 | 5614.12 | Euploid |  |
| 193.66 | 150 | 1.33 | 171.45 | 5046.87 | Euploid |  |
| 208.38 | 177 | 1.35 | 125.65 | 5213.24 | Euploid |  |
| 196.42 | 159 | 1.39 | 114.83 | 6626.73 | Euploid |  |
| 162.61 | 60 | 0.78 | 443.40 | 6281.66 | Mosaic | (-X, 60%) |
| 181.01 | 96 | 0.98 | 172.14 | 4869.13 | Euploid |  |
| 179.76 | 141 | 1.43 | 429.47 | 2933.73 | Euploid |  |
| 152.32 | 90 | 1.29 | 1514.24 | 3019.97 | Aneuploid | +9q; -21; -22 |
| 192.08 | 75 | 0.66 | 924.69 | 2147.17 | Aneuploid | -8; -10 |
| 189.52 | 135 | 1.26 | 175.78 | 5432.82 | Euploid |  |
| 196.88 | 72 | 0.61 | 564.30 | 4099.86 | Aneuploid | +15; -18; -22 |
| 195.27 | 144 | 1.25 | 122.98 | 4618.52 | Euploid |  |
| 198.72 | 80 | 0.66 | 566.07 | 3743.68 | Mosaic | (-19p13.3p13.2, 60%) |
| 177.33 | 84 | 0.90 | 331.23 | 5137.75 | Aneuploid | -21; (-8q22.2q24.3, 30%) |
| 191.36 | 162 | 1.48 | 123.98 | 5665.01 | Euploid |  |
| 169.74 | 48 | 0.56 | 2104.44 | 5513.03 | Aneuploid | -16 |
| 169.05 | 96 | 1.16 | 167.48 | 7018.19 | Euploid |  |
| 183.08 | 99 | 0.98 | 175.87 | 4710.37 | Euploid |  |
| 210.68 | 38 | 0.28 | 636.01 | 4029.55 | Aneuploid | -16; (+7q11.1q21.11, 35%); (-Xp22.33p22.2, 35%) |
| 135.47 | 72 | 1.32 | 153.61 | 3283.24 | Euploid |  |
| 170.66 | 158 | 1.85 | 107.02 | 6239.50 | Euploid |  |
| 157.55 | 78 | 1.03 | 275.01 | 2616.06 | Mosaic | (-3, 30%); (-4, 30%) |
| 172.04 | 162 | 1.80 | 104.13 | 2889.19 | Aneuploid | -X |
| 186.30 | 62 | 0.58 | 329.52 | 1232.15 | Euploid |  |
| 165.60 | 114 | 1.39 | 135.70 | 4338.96 | Euploid |  |
| 175.49 | 134 | 1.46 | 113.79 | 5022.64 | Euploid |  |
| 198.72 | 132 | 1.11 | 137.23 | 4782.21 | Euploid |  |
| 159.62 | 58 | 0.76 | 172.90 | 3914.86 | Mosaic | (-X, 30%) |
| 183.54 | 120 | 1.17 | 223.15 | 3000.75 | Euploid |  |
| 165.83 | 75 | 0.91 | 303.00 | 3830.33 | Mosaic | (+16, 30%); (+10, 20%) |
| 176.18 | 93 | 0.97 | 156.52 | 1730.62 | Euploid |  |
| 173.19 | 144 | 1.61 | 119.01 | 4836.81 | Aneuploid | +15 |
| 191.59 | 39 | 0.35 | 373.26 | 4830.35 | Euploid |  |
| 175.49 | 149 | 1.61 | 150.95 | 4268.75 | Euploid |  |
| 165.60 | 78 | 0.93 | 275.93 | 2635.11 | Mosaic | (-1q23.1q44, 35%); (+9p13.3p11.2, 30%) |
| 196.42 | 99 | 0.87 | 154.95 | 6768.24 | Euploid |  |
| 201.02 | 156 | 1.28 | 117.83 | 5076.34 | Euploid |  |
| 182.85 | 147 | 1.44 | 176.95 | 3207.33 | Euploid |  |
| 213.90 | 219 | 1.60 | 115.38 | 7163.29 | Euploid |  |
| 218.50 | 112 | 0.78 | 257.78 | 6329.33 | Mosaic | (+2q32.1q37.3, 30%); (+8, 30%) |
| 193.20 | 204 | 1.85 | 90.42 | 7000.47 | Euploid |  |
| 212.06 | 150 | 1.11 | 160.99 | 6214.37 | Euploid |  |
| 188.37 | 147 | 1.36 | 125.53 | 3303.98 | Euploid |  |
| 178.02 | 86 | 0.93 | 385.46 | 7460.75 | Aneuploid | -15; -17 |
| 184.00 | 58 | 0.57 | 504.82 | 4441.33 | Aneuploid | +16 |
| 170.20 | 50 | 0.58 | 407.59 | 4206.24 | Aneuploid | +8; +21 |
| 183.08 | 156 | 1.53 | 340.44 | 3541.44 | Mosaic | (+14q11.2q23.2, 65%); (-14q23.2q32.33, 40%) |
| 167.67 | 83 | 0.99 | 312.41 | 4804.11 | Aneuploid | -X; (+16, 30%); (+22, 30%) |
| 183.54 | 76 | 0.75 | 642.29 | 4529.88 | Aneuploid | -14 |
| 197.34 | 192 | 1.67 | 110.85 | 7475.14 | Euploid |  |
| 199.53 | 198 | 1.70 | 136.76 | 8423.79 | Euploid |  |
| 204.01 | 255 | 2.06 | 90.60 | 6745.81 | Euploid |  |
| 172.27 | 122 | 1.39 | 313.48 | 5359.25 | Euploid |  |
| 192.51 | 138 | 1.22 | 292.36 | 3606.722 | Euploid |  |
| 205.62 | 180 | 1.42 | 231.10 | 6279.07 | Euploid |  |
| 191.52 | 151 | 1.38 | 246.93 | 5863.45 | Euploid |  |
| 179.40 | 117 | 1.24 | 225.82 | 7026.07 | Euploid |  |
| 214.82 | 193 | 1.38 | 202.82 | 5133.36 | Euploid |  |
| 209.76 | 189 | 1.44 | 144.87 | 7261.74 | Euploid |  |
| 183.08 | 131 | 1.30 | 223.87 | 4644.99 | Euploid |  |
| 181.93 | 89 | 0.90 | 245.58 | 5060.52 | Mosaic | (-21, 30%) |
| 180.32 | 134 | 1.38 | 165.74 | 5078.88 | Euploid |  |
| 176.41 | 161 | 1.73 | 119.71 | 4682.23 | Euploid |  |
| 158.93 | 96 | 1.25 | 153.13 | 2573.64 | Euploid |  |
| 191.59 | 82 | 0.73 | 341.94 | 3557.74 | Aneuploid | -4 |
| 164.45 | 66 | 0.81 | 523.73 | 3623.28 | Mosaic | (-15, 30%) |
| 171.35 | 64 | 0.72 | 409.63 | 3818.43 | Aneuploid | -13; -15 |
| 195.27 | 64 | 0.57 | 487.40 | 7093.73 | Aneuploid | -15 |
| 180.78 | 72 | 0.72 | 322.54 | 2513.07 | Aneuploid | -19 |
| 143.75 | 48 | 0.82 | 347.50 | 6471.26 | Aneuploid | +2 |
| 209.30 | 110 | 0.80 | 299.58 | 0.00 | Aneuploid | -13; +15 |
| 202.86 | 156 | 1.25 | 203.66 | 4541.20 | Euploid |  |
| 218.73 | 123 | 0.85 | 227.16 | 4891.77 | Euploid |  |
| 189.98 | 106 | 0.97 | 473.29 | 4295.64 | Mosaic | (+21, 30%) |
| 221.03 | 147 | 1.01 | 145.52 | 7484.93 | Euploid |  |
| 178.94 | 82 | 0.85 | 275.03 | 4412.55 | Mosaic | (-2, 40%) |
| 201.02 | 171 | 1.40 | 126.56 | 5156.22 | Euploid |  |
| 203.32 | 153 | 1.23 | 140.56 | 4979.74 | Euploid |  |
| 198.03 | 141 | 1.19 | 138.80 | 5086.49 | Euploid |  |
| 212.29 | 198 | 1.47 | 157.03 | 6784.69 | Euploid |  |
| 181.01 | 120 | 1.21 | 143.32 | 3635.76 | Euploid |  |
| 167.44 | 72 | 0.86 | 289.59 | 4817.13 | Aneuploid | +19 |
| 161.46 | 102 | 1.30 | 78.59 | 3603.65 | Euploid |  |
| 206.08 | 64 | 0.49 | 518.60 | 3396.02 | Aneuploid | -22 |
| 209.30 | 243 | 1.81 | 96.97 | 3296.99 | Euploid |  |
| 105.11 | 40 | 1.15 | 324.40 | 0.00 | Aneuploid | -7; +16; -22 |
| 114.54 | 48 | 1.25 | 247.52 | 2749.21 | Aneuploid | +4; +5 |
| 174.11 | 70 | 0.79 | 508.78 | 6657.41 | Aneuploid | -10 |
| 177.56 | 64 | 0.66 | 326.58 | 2798.41 | Aneuploid | -15 |
| 199.41 | 132 | 1.11 | 247.29 | 6528.55 | Aneuploid | -14 |
| 194.35 | 186 | 1.64 | 107.45 | 5110.09 | Euploid |  |
| 192.51 | 108 | 0.99 | 412.43 | 6923.29 | Mosaic | (+2, 50%); (+4, 50%); (+13, 50%); (+15, 50%); (+20, 50%); (+21, 50%) |
| 142.60 | 57 | 0.95 | 219.47 | 3981.20 | Aneuploid | -3 |
| 204.24 | 66 | 0.52 | 793.95 | 5157.27 | Aneuploid | -13 |
| 137.54 | 51 | 0.92 | 378.24 | 4284.37 | Aneuploid | +22; (+3, 50%); (+6, 50%); (+7, 50%); (+20, 50%); (+X, 50%) |
| 168.13 | 60 | 0.72 | 538.69 | 4893.67 | Aneuploid | +9; +14 |
| 191.82 | 102 | 0.92 | 224.47 | 5101.62 | Euploid |  |
| 198.26 | 138 | 1.16 | 178.62 | 4130.43 | Euploid |  |
| 174.57 | 102 | 1.11 | 190.85 | 3635.55 | Euploid |  |
| 196.65 | 153 | 1.31 | 198.93 | 4949.91 | Euploid |  |
| 171.35 | 111 | 1.27 | 154.78 | 4898.70 | Euploid |  |
| 173.42 | 114 | 1.27 | 217.34 | 4756.08 | Euploid |  |
| 166.52 | 72 | 0.91 | 198.67 | 8368.36 | Euploid |  |
| 169.51 | 108 | 1.26 | 200.16 | 4476.45 | Euploid |  |
| 208.15 | 70 | 0.53 | 513.35 | 4880.29 | Aneuploid | -20; +21 |
| 159.16 | 89 | 1.20 | 144.73 | 5544.34 | Aneuploid | -22 |
| 160.54 | 88 | 1.16 | 257.00 | 5112.26 | Aneuploid | -22 |
| 175.26 | 70 | 0.75 | 303.43 | 2871.25 | Aneuploid | +16 |
| 157.55 | 76 | 1.03 | 145.97 | 4174.29 | Aneuploid | -21 |
| 138.69 | 36 | 0.65 | 794.62 | 5170.02 | Mosaic | (-18, 50%) |
| 121.90 | 69 | 1.66 | 156.10 | 5107.39 | Euploid |  |
| 174.57 | 120 | 1.30 | 135.35 | 3238.96 | Euploid |  |
| 179.40 | 135 | 1.39 | 116.96 | 4315.95 | Euploid |  |
| 181.70 | 90 | 0.90 | 522.85 | 4059.07 | Aneuploid | -14 |
| 182.62 | 139 | 1.40 | 180.87 | 5618.46 | Euploid |  |
| 195.27 | 172 | 1.50 | 165.78 | 5186.69 | Euploid |  |
| 184.92 | 117 | 1.13 | 368.15 | 4199.73 | Euploid |  |
| 174.80 | 158 | 1.73 | 197.13 | 4717.78 | Euploid |  |
| 207.00 | 175 | 1.35 | 273.14 | 4938.53 | Euploid |  |
| 151.57 | 123 | 1.81 | 165.21 | 4062.14 | Euploid |  |
| 169.05 | 66 | 0.77 | 288.55 | 4401.86 | Aneuploid | +8; +12 |
| 207.69 | 198 | 1.53 | 97.78 | 6092.49 | Euploid |  |
| 158.01 | 76 | 1.04 | 258.78 | 5377.55 | Aneuploid | +7; (+1, 50%); (-6, 30%); (+9, 30%); (+10, 30%); (+11, 30%); (+14, 30%); (+22, 30%) |
| 191.82 | 132 | 1.21 | 201.94 | 6898.95 | Euploid |  |
| 182.85 | 117 | 1.16 | 293.36 | 3751.40 | Euploid |  |
| 180.55 | 78 | 0.79 | 298.59 | 4036.90 | Aneuploid | -16; (+3p26.3p12.3, 30%) |
| 190.67 | 219 | 2.03 | 89.63 | 6144.23 | Euploid |  |
| 125.35 | 44 | 0.96 | 318.72 | 3313.13 | Mosaic | (-10,35%) |
| 183.31 | 78 | 0.77 | 317.02 | 3970.15 | Aneuploid | (-10q23.32q26.3, 30%) |
| 158.93 | 119 | 1.58 | 264.75 | 3975.76 | Euploid |  |
| 193.66 | 177 | 1.55 | 157.43 | 3274.67 | Euploid |  |
| 167.21 | 70 | 0.85 | 341.28 | 5618.14 | Aneuploid | +16p13.3p11.1; (-7q11.1q36.3, 30%) |
| 178.25 | 82 | 0.86 | 318.56 | 4793.64 | Aneuploid | -8 |
| 180.55 | 70 | 0.71 | 521.49 | 4314.63 | Aneuploid | -21; -22 |
| 136.62 | 40 | 0.72 | 723.35 | 3277.47 | Mosaic | (+1,50%); (+9,35%); (+13,30%) |
| 141.95 | 35 | 0.62 | 594.26 | 6492.86 | Aneuploid | -14; (-16, 30%) |
| 165.37 | 38 | 0.50 | 537.67 | 9219.99 | Aneuploid | -17q; -Xq21.2q28 |
| 206.77 | 168 | 1.33 | 135.93 | 7774.34 | Euploid |  |
| 170.66 | 62 | 0.72 | 321.76 | 5038.73 | Aneuploid | +9; +14 |
| 201.02 | 194 | 1.59 | 267.86 | 4963.82 | Euploid |  |
| 182.16 | 109 | 1.11 | 412.79 | 5674.29 | Euploid |  |
| 203.78 | 218 | 1.77 | 195.79 | 7592.46 | Euploid |  |
| 178.02 | 72 | 0.76 | 286.89 | 5230.38 | Aneuploid | +1 |
| 188.14 | 114 | 1.07 | 174.51 | 4480.05 | Euploid |  |
| 198.03 | 168 | 1.44 | 130.09 | 6195.49 | Euploid |  |
| 204.70 | 229 | 1.86 | 105.61 | 8673.38 | Euploid |  |
| 183.77 | 64 | 0.63 | 332.48 | 4368.06 | Mosaic | (+3, 35%); (+5, 30%); (+6, 30%); (+7, 50%); (+12, 45%); (+15, 40%);(-21, 40%); (+22, 30%) |
| 189.52 | 148 | 1.39 | 276.49 | 6015.33 | Euploid |  |
| 183.08 | 189 | 1.87 | 103.79 | 4268.34 | Euploid |  |
| 204.70 | 82 | 0.65 | 727.36 | 5048.35 | Mosaic | (+6, 40%) |
| 192.51 | 147 | 1.30 | 246.84 | 3336.98 | Euploid |  |
